# Cryopreservation of GABAergic Neuronal Precursors for Cell-Based Therapy

**DOI:** 10.1371/journal.pone.0170776

**Published:** 2017-01-25

**Authors:** Daniel Rodríguez-Martínez, María Magdalena Martínez-Losa, Manuel Alvarez-Dolado

**Affiliations:** Laboratory of Cell-based Therapy for Neuropathologies, Andalusian Center for Molecular Biology and Regenerative Medicine (CABIMER), CSIC, Seville, Spain; Faculty of Animal Sciences and Food Engineering, University of São Paulo, BRAZIL

## Abstract

Cryopreservation protocols are essential for stem cells storage in order to apply them in the clinic. Here we describe a new standardized cryopreservation protocol for GABAergic neural precursors derived from the medial glanglionic eminence (MGE), a promising source of GABAergic neuronal progenitors for cell therapy against interneuron-related pathologies. We used 10% Me_2_SO as cryoprotectant and assessed the effects of cell culture amplification and cellular organization, as *in toto* explants, neurospheres, or individualized cells, on post-thaw cell viability and retrieval. We confirmed that *in toto* cryopreservation of MGE explants is an optimal preservation system to keep intact the interneuron precursor properties for cell transplantation, together with a high cell viability (>80%) and yield (>70%). Post-thaw proliferation and self-renewal of the cryopreserved precursors were tested *in vitro*. In addition, their migration capacity, acquisition of mature neuronal morphology, and potency to differentiate into multiple interneuron subtypes were also confirmed *in vivo* after transplantation. The results show that the cryopreserved precursor features remained intact and were similar to those immediately transplanted after their dissection from the MGE. We hope this protocol will facilitate the generation of biobanks to obtain a permanent and reliable source of GABAergic precursors for clinical application in cell-based therapies against interneuronopathies.

## Introduction

Interneuron-related pathologies (interneuronopathies) comprise a wide and relevant group of diseases, including epilepsy, schizophrenia, infantile encephalopathies, autism spectrum disorder, or Alzheimer’s disease [1–5]. In the last years, different groups have been working in innovative cell-based therapeutic approaches to treat this group of neuropathologies [6–9]. Very promising results have been achieved grafting GABAergic interneuron precursors derived from the MGE, the subpallial region of the embryonic brain where most of the cortical interneurons are generated during development [10,11]. This therapeutic strategy has led to reversion of symptomatology in multiple animal models of the above mentioned diseases [6–9].

Hitherto, the transplants were performed isolating the MGE-derived precursors from E12-E14 mouse embryos, following on mechanical dissociation, and immediate grafting into the neonatal or adult brain, with no culture or any further manipulation. After transplantation, these precursors spread out and cover wide areas of the cortex, striatum and hippocampus [12]. They can migrate several mm during the first week, to later stop and acquire the morphology of mature interneurons. Four weeks after transplantation they have fully differentiated, expressing GABA and specific interneuron subtype markers such as, parvalbumin, somatostatin, calretining, or NP-Y. Their proportions are similar to those normally generated by the MGE during development [12,13], in concordance with their intrinsically determined differentiation program [14]. It has been shown they integrate in the host circuitry and are able to modify the cortical and hippocampal inhibitory tone [12,13]. Moreover, electron microscopy, electrophysiological analysis of spontaneous and evoked synaptic currents, and simultaneous electrode recordings of transplanted interneurons and host projection neurons have shown they form functional inhibitory synaptic connections [12,15,16]. Finally, these precursors present a good long term survival rate (around 15% a year and a half after transplantation in the mouse brain) with no side effects such as gliosis, or tumor formation, what points to their high safety standard [6]. All these properties make the MGE-derived GABAergic precursors the most promising neuronal progenitor for cell-based therapies against interneuronopathies.

To apply these precursors in the clinical setting it would be necessary a permanent source of cells ready for transplantation. The establishment of biobanks, in where to store MGE-derived cells from altruist donations, should facilitate the provision of cells ready for their immediate use. An alternative would be the generation of GABAergic precursors from induced pluripotent stem cells (iPSC). Several groups have reported driven differentiation and transplantation of iPSC-derived interneurons in animal models of epilepsy with promising results [17,18]. In any case, these cell cultures would need somehow to be stored as well. To our knowledge, currently there is no description of a specific preservation system for this type of interneuron precursors. Therefore, it is crucial to set up an efficient cryopreservation protocol to properly collect these GABAergic precursors, preserving always their unique intrinsic features, and so facilitating their clinical application.

Cryopreservation is the process by which cells or tissues are frozen at very low temperatures, generally between -80°C and -196°C, to reduce cellular functions and keep life suspended [19]. It is a controlled process of reversible cellular dehydration and enzyme activity suspension that allows cell storage for very long periods. There are different methods of cryopreservation that mainly differ in their freezing/thawing rates [19,20], use of cryoprotectants [21], and cell density [19,20,22]. Modification of these parameters makes a protocol more suitable for a cell type than to another. Dimethyl sulfoxide (Me_2_SO), the most extendedly used intra-cellular cryoprotectant, has been reported to maintain multipotency and render excellent viability of murine neural progenitors when applied at 7–10% [23]. Based on the Me_2_SO usage background and the previous literature about nervous tissue and neural progenitor cryopreservation [24–29], we considered this crioprotectant as likely suitable to preserve MGE-derived GABAergic neuronal precursors. Regarding cooling rates, there are two general methods. Slow cooling rate at 1°C/min that tries to reduce cell membrane damage due to the formation of ice crystals [19]; and vitrification that aims to avoid ice crystal formation on both cooling and warming by achieving glass-like solidification [20]. Several works have shown that both methods are suitable for nervous tissue and there are no differences between slow cooling and vitrified neural progenitor cells in terms of stemness marker expression, proliferation, or multipotent differentiation [30,31]. Finally, cell density or tissue structure is also an important factor to take into account. Successful cryopreservation of brain tissue, neurospheres (NS), or individually dissociated neurons may diverge due to their different structure and permeability to cryoprotectants [22,32]. Freezing of whole explants from different brain areas has been a common practice in transplantation research field. The results suggest that preservation of the complete structure may be beneficial for cellular integrity and to keep neuronal properties. In this regard, several reports indicate that murine brain explants cryopreserved as a whole structure (*in toto*) show comparable properties to the same tissues grafted immediately after resection from the donor [27,28,33–35]. However, in the case of GABAergic neuronal precursors, analysis of the tissue structure effect on their post-thaw cellular viability remains to be addressed.

The present work analyses in-depth the cryopreservation efficiency when using different physical states of MGE-derived precursors. And, as a result, we propose the best procedure to keep unaltered the migratory, proliferative and differentiation properties of these precursors after cryopreservation in order to apply them in regenerative medicine. To avoid complexity, we based our procedure in slow cooling rates and Me_2_SO as cryoprotectant, given their good results in neuronal stem cell cryopreservation [24–29]. We assessed different conditions of cellular density and physical complexity: dissociated cells; NS; or whole MGE tissue (*in toto* condition). Finally, we also analyzed the effects of an intermediate step of culture amplification as NS, since we consider of interest to obtain a higher amount of precursors prior to their storage and transplantation. Our results show that fresh isolated *in toto* MGE explants, instead of dissociated or cultured cells, render optimal results in viability, survival, proliferation and differentiation of the MGE-derived GABAergic precursors.

## Materials and Methods

### Experimental animals

CD-1 adult mice (60 days old) were obtained from Charles River (Barcelona, Spain) and kept in the CABIMER animal facility with water and food provided *ad libitum*. For transplantation assays we bred GFP^+^ transgenic males [36] (in CD1 background) with wildtype CD1 females to obtain fluorescent embryos as MGE cell donors, whereas the receptors were wildtype adult CD1 mice. To obtain MGE tissue mice were mated overnight, and the presence of a vaginal plug was considered as embryonic day 0.5 (E0.5). Pregnant females were not disturbed, except for weekly cage cleaning, until E12.5, when they were euthanized by cervical dislocation and embryos dissected for MGE isolation. Experimental animals were sacrifized by anesthesia overdose before to be transcardially perfused with fixatives. All experiments were conducted in accordance with the European (Directive 2010/63/UE) and Spanish (Real Decreto 53/2013, Ley 32/2007 and 9/2003) regulations for animal research. Experimental protocols were approved by the Comité Ético de Experimentación Animal of CABIMER (9/2009) and authorized by the Dirección General de la Producción Agrícola y Ganadera de la Consejería de Agricultura, Pesca y Medio Ambiente of the Junta de Andalucía (450/10112).

### MGE isolation and dissociation

The mouse MGE is easily distinguished from other subpallial structures at E12-E13. At this age, generation of GABAergic precursors is at its maximum and a sulcus clearly separate MGE from LGE, facilitating a clean dissection. Each embryo´s brain was dissected in cold Dulbecco’s Modified Eagle’s Medium/F-12 (DMEM/F12; mixture 1:1; Gibco^®^ by life technologies^™^, Madrid, Spain) and MGE explants collected in eppendorf tubes. For *in toto* cryopreservation condition the explants were straight away frozen at this point. For further dissociation, the tissue was mechanically disaggregated by pipetting up-down in presence of DNase I (1000 U/ml Roche, USA). Cell suspension was washed and centrifuged to be resuspended in DMEM/F12 medium.

### Cryopreservation protocols

**Fig 1:**
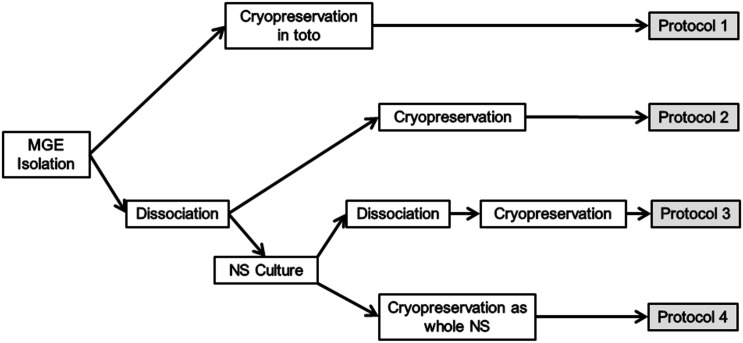
Procedure scheme followed for each cryopreservation protocol.

We established four different cryopreservation procedures in order to test different physical states of the MGE-derived precursors (Fig 1):

1. Fresh isolated *in toto* MGE explants frozen in cryopreservation solution (protocol-1)
2. Fresh dissociated MGE-derived cells frozen in cryopreservation solution (protocol-2)
3. Fresh isolated MGE-derived cells cultured as NS for amplification and then dissociated as individual cells before being frozen in cryopreservation solution (protocol-3)
4. Fresh isolated MGE-derived cells cultured as NS for amplification and frozen intact as spheres in cryopreservation solution (protocol-4).

We started all the protocols with a similar number of cells (3.5x10^6^ cells/cryovial). This number of cells corresponds to seven GFP^+^ embryos, a mean estimation obtained from previous results of our laboratory (Data not shown). We performed at least 5 cryopreservation assays for each tested protocol. In protocols 1 and 2 we immediately freeze the cells or explants in cryopreservation solution (10% Me_2_SO in DMEM/F12 medium) after the dissection procedure. For protocols 3 and 4 MGE-derived cells were cultivated after their isolation and amplified in complete defined medium until the formation of NS (see next section). After a 1:1 passage, new NS were formed and half of them were mechanically dissociated and frozen in cryopreservation solution for protocol-3, whereas the other half was directly frozen as NS in cryopreservation solution for protocol-4.

For all protocols, samples (explants, dissociated cells and NS) were resuspended in cryopreservation solution and the vials quickly transferred to a refrigerator at -80°C into a Nalgene^™^ Mr. Frosty Freezing Container that provides repeatable -1°C/minute cooling rate according to the manufacturer. One day later, vials were transferred to a liquid nitrogen container for long term storage in vapor phase nitrogen. One month after cryopreservation vials from each protocol were quickly thawed in a warm bath at 37°C, washed, and resuspended in DMEM/F12 medium for further analysis.

### Neurosphere cultures

Cell suspension obtained after MGE dissociation or after thawing was plated in special P35 plates for cell suspension culture (Sarstedt, Nümbrecht, Germany) at a density of 0.5-1x10^6^ cells/ml in the presence of serum-free expansion complete medium: DMEM/F12 mixture (1:1) supplemented with 0.6% Glucose, 0.11% NaHCO3, 2 mM L-glultamine, 100U/ml /0.250 μg/ml Antibiotics/Antimicotics, 10% Hormone Mix, 20 ng/ml of Human Recombinant Epidermal Growth Factor (EGF) (Peprotech, Neuilly-Sur-Seine, France), and 10 ng/ml Fibroblast Growth Factor (FGF) (PromoCell, Heidelberg, Germany). Plates were incubated at 37°C in 5% CO2 atmosphere. Every 3 days in culture, expansion complete medium was added and the culture status and NS formation was verified.

### MGE-derived GABAergic progenitor adhesion culture

MGE-in toto tissue frozen following Protocol -1 and obtained from one litter (~10 embryos) was thawed and plated on 1% gelatin-coated flasks (25 cm^2^, SPL Life Sciences, Gyeonggi-do, Korea) in serum-free expansion complete medium. Medium was changed every 3–4 days. After 4–6 weeks, once the flasks were ~ 90% confluent, cells were subculture to 0.1% gelatin-coated TC coverslips (13 mm diameter, Sarstedt, Nümbrecht, Germany) in 24 well plates (Corning Inc., Fisher Scientific, Madrid, Spain) at 2 x 10^5^ cells/ml in serum-free expansion complete medium. Medium was changed every 3–4 days. Immunocytochemistries were performed on TC coverslips at confluency.

### Cell viability and yield

Before and after cryopreservation, a small cell aliquot from each protocol was suspended (1:1) in Trypan Blue 0.2% solution (Gibco-Invitrogen, USA) and counted in a Neubauer chamber. Viability was expressed as the percentage of unstained cells (alive) in the total counted population. In order to assess the final recovery of cells after crypreservation we measured the cellular yield, expressed as 100 _*_ (number of post-thaw recovered cells / initial number of cells).

### Transplantation

Intracerebral administration of cryopreserved GFP^+^ GABAergic progenitors was performed with beveled glass micropipettes (50–70μm diameter, Drummond Scientific, USA) coupled to a stereotaxic apparatus and controlled with the help of a microinjector (MO-10, Narishige, Tokyo, Japan), as previously described [12,13]. Cells were pelleted by centrifugation (5 min, 300xg) and resuspended in DMEM/F12 at 0.1–0.5x10^6^ cells/μl. MGE cell suspension were loaded into glass micropipettes prefilled with pure mineral oil and then with DMEM/F12.

Transplants were performed in neonatal mice (P3-P5) as previously described [12]. Mice were anesthetized by hypothermia until pedal reflex was abolished (approximately 1–2 minutes). Anesthesia was maintained by performing surgery on a cold aluminum plate. We used the following stereotaxic coordinates from Bregma (according to the Atlas of Developing Mouse Brains E17, 5, P0 and P6. Paxino, George / Halliday) for cortex (2.2mm A, 3.5 mm L, 1.2 mm D), and hippocampus (-1.2 mm A, 1.7 mm L, 2.0 mm D). After surgical procedure, grafted mice were returned to their cages and analyzed one month later.

### Immunostaining and cell counting

Tissue processing and immunohistochemistry were performed as previously described [12,13]. Briefly, mice were transcardially perfused with 4% PFA in 0.1 M PBS and, after overnight postfixation in the same fixative, brains were coronally sectioned into six series of 50 μm slices using a vibratome (Leyca). Free floating immunostainings were performed with the following antibodies: mouse anti-parvalbumin (1:1000, 235 Swant, Marly, Switzerland), rabbit anti-calbindin 28K (1:2000, CB-38a Swant, Marly, Switzerland), rabbit anti-calretinin (1:200, 7699/4 Swant, Marly, Switzerland), rabbit anti-somatostatin28 (1:500, NB 100–64650 Novus Biologicals, Cambridge, UK), rabbit anti-neuropeptide Y (1:500, N9528 Sigma, Madrid, Spain), mouse anti-GFP (1:1000, MAB2510 Merck Millipore, Darmstadt, Germany), rabbit anti-GFP (1:3000, Ab290 Abcam, Cambridge, UK). Secondary antibodies: Rhodamine Red Anti-rabbit, Rhodomine Red Anti-mouse, Fluorescein Anti-rabbit and Flouresecein Anti-mouse (all 1:400, Jackson ImmunoResearch, Suffolk, UK). Biotinylated secondary antibodies and ABC kit (Vector Laboratories, Peterborough, UK) were used for peroxidase reaction with diaminobenzidine to detect GFP. As negative controls primary antibodies were omitted from the reaction in the presence of all the reagents and secondary antibodies. All the negative controls were run in parallel for each immunodetection assay and always resulted in lack of staining.

Inmunocytofluorescence of MGE-derived GABAergic precursors cultured on 0.1% gelatine-coated TC coverslips was performed as follows. Cell cultures were fixed with 2% paraformaldehyde for 10 minutes at RT, followed by two washes in 0.1 M PBS. Blocking was performed for 1 hour in 0.1 M PBS/10% Normal Goat Serum (NGS) / 0.1% Triton X-100 at RT. Primary antibody was diluted in blocking solution and incubated overnight at 4°C in continuous motion. After washing with PBS the secondary antibody was added and incubated at RT for 1 hour in the same blocking solution. The following antibodies were used: Mouse anti-Nestin (1:100, Chemicon, Merck Millipore, Darmstadt, Germany), Mouse anti-Map2 (1:100, Sigma, Madrid, Spain), Mouse anti-NeuN (1:100, Merck Millipore, Darmstadt, Germany), Rabbit anti-GFAP (1:100, Dako, Barcelona, Spain), Rabbit anti-Olig2 (1:100, Chemicon, Merck Millipore, Darmstadt, Germany) and Rabbit anti-NG2 (1:100, Sigma, Madrid, Spain). Secondary antibodies: FITC anti-mouse and Cy-5 anti-rabbit (both at 1:400, from Jackson ImmunoResearch, Suffolk, UK). Negative controls, in wich primary antibodies were omitted, resulted in lack of staining. Nuclei were stained with 4′,6-diamidino-2-phenylindole (DAPI).

Quantification of double positive cells for GFP and interneuron markers was performed on images obtained with a digital Leica DFC350 FX camera coupled to a Leica AF6000 microscope (Leica Microsystems, Germany) and specific LAS AF software (Leica Microsystems Imaging Solutions, Cambridge, UK). At least 200 GFP^+^ cells were analyzed for every marker and animal. The survival rate of the cells at 4 weeks after transplantation was estimated in serial slices counting all GFP^+^ cells in the temporal septum—anterior- posterior axis (7 coronal sections of 50μm separated 300μm between them, covering injection site and a region from—1.06mm to—3.28mm along the longitudinal axis from Bregma). The survival rate was estimated from the ratio 100 x (number of surviving cells) / number of transplanted cells (5x10^4^). Statistical comparison between groups was calculated using unpaired Student 's t-test with significance level of p < 0.05.

### Morphological analysis (Sholl analysis)

Sholl analysis was performed by using a software plugin installed in Image J (1.46r) NIH software: (Advance Sholl Analysis, Ferreira and Maddock, http://imagej.net/Sholl_Analysis). This methodology creates a series of concentric circles around the soma of the neuron and several parameters can be obtained measuring how the neuronal arbor interact with them [37]. Photomicrographs from fresh isolated (n = 35) and cryopreserved (n = 36) cells transplanted into the somatosensory cortex (n = 8) were analyzed overdrawing circles spaced at 10μm intervals from the cell soma. Dendritic intersections and the following parameters were measured to give us a complete morphological study of the neurons: the area under the curve representing the number of intersections N versus the radius (r, μm), which gives us an approximation of the dendritic tree extension and shape; the mean value of function (Nav), representing the average number of intersections along the function; the maximum density of intersections (Nm); the maximal distance to the last concentric circle from the soma (Nr); the slope of the linear regression from the Sholl decay plot (K), that represents the rate of decay of the number of branches with the distance; and finally the ratio between Nm and the number of primary branches that interact with the first circle (RI). Statistical comparison between groups was calculated applying unpaired Student 's t-test with a significance level of p < 0.05.

## Results

In order to establish the best cryopreservation conditions for MGE-derived GABAergic precursors we designed four different experimental procedures. We assessed how the physical structure (*in toto* explants, NS, or dissociated cells) affected the cell viability of frozen MGE-derived precursors. In addition, we studied the influence of an *in vitro* amplification step on their survival and features. Detailed description of the four experimental protocols can be found in materials and methods section (Fig 1).

### Cell viability and yield

Cell survival was analyzed by trypan blue staining before and after cryopreservation process. Prior to freezing the percentage of viable fresh-dissociated cells in protocol-2 was 90.5 ± 0.7%. For *in toto* condition (protocol-1) is not possible to directly measure in the explants their cell viability. Thus, for later calculations we considered similar viability values to protocol-2. Nonetheless, this is an underestimation, since mechanical dissociation performed in protocol-2 may lead to cell loss. For protocols-3 and 4 the cell viability values were 89.14 ± 1.7% and 91.65 ± 1.8%, respectively. These high viability values show MGE-derived cells were in optimal condition after their isolation and prior to cryopreservation. One month after cryopreservation process (see M&M section), cryovials were quickly thawed in a warm bath at 37°C, washed in DMEM/F12 medium, and cell viability was immediately determined. Table 1 shows the viability results for each protocol. We observed small, not statistically significant, differences in cell viability among the protocols. Viability values were around 80 ± 10%, similar to or above previously reported cryopreservation protocols for neural cells or tissue [27–29,34]. However, we observed an important reduction in the number of retrieved cells with some of the protocols. Quantifications for the recovery yield (100 _*_ number of post-thaw recovered cells / initial number of cells) in each procedure are shown in Table 1.

**Table 1:** Quality of the tested cryopreservation protocols.

	Pre-cryoViability	Post-cryoViability	RecoveryYield	Ability togenerate NS
**Protocol-1**	N/D	76,90 ± 2,4%	71,44 ± 7,2%	+++
**Protocol-2**	90,51 ± 0,7%	66,47 ± 12%	59,32 ± 14,4%	-
**Protocol-3**	91,65 ± 1,8%	75,43 ± 5,8%	34,36 ± 4,5%	+
**Protocol-4**	89,14 ± 1,8%	92,23 ± 7,3%	29,60 ± 5,4%	-

Cell survival was determined by tripan blue staining for each cryovial (n = 5). Data represented as mean ± SEM. After cell plating to form NS, the cultures were classified as: (-) not able to form NS; (+) slow formation of primary NS; (+++) fast and vigorous formation of primary and secondary NS. N/D; Not determined.

**Fig 2:**
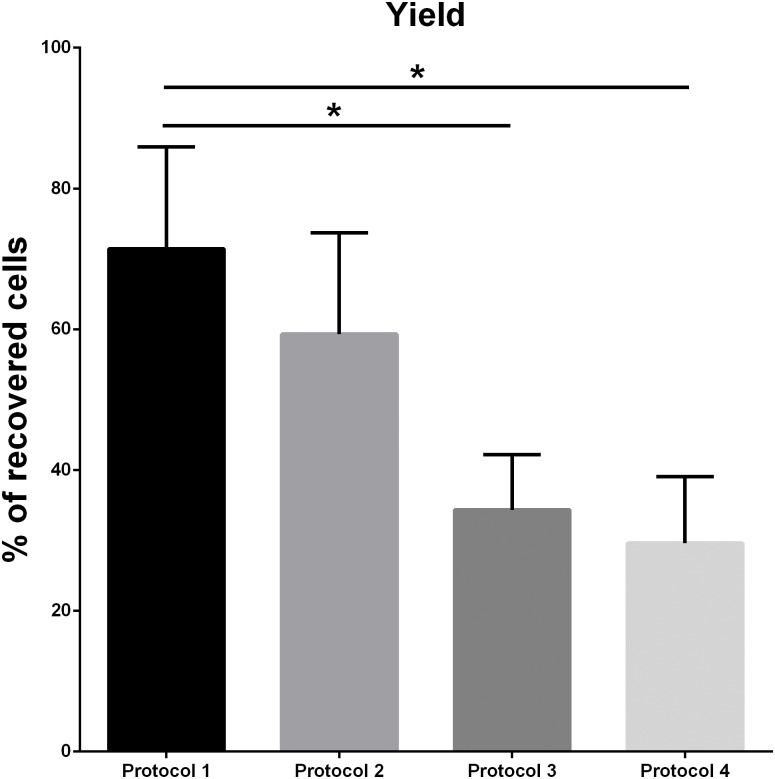
Cell recovery yield after cryopreservation. Representation of the retrieved cell percentage for each cryopreservation protocol immediately after thawing. Values expressed as mean + SEM (n = 5). Statistical analysis: One-way ANOVA with Tukey's multiple comparison post-hoc test (* = p<0.05).

Data reveled that recovery yields for protocols-1 and 2 were twofold higher than for protocols 3 and 4 (Fig 2). Thus, whereas *in toto* MGE cryopreservation renders a 70% yield approximately, we only recover one third of the cells with protocols-3 and 4. This result strongly suggests that MGE-derived cells that were cultured *in vitro* are later more susceptible to cell death and lysis during cryopreservation process.

### *In vitro* characterization of post-thawed cells

**Fig 3:**
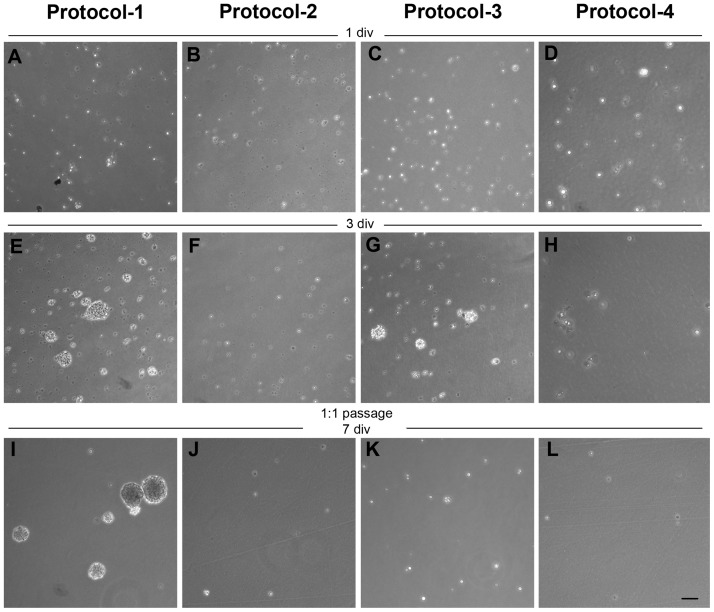
NS cultures of MGE-derived precursors after cryopreservation. (A-D) Phase-contrast photomicrographs of thawed cells plated 1 day *in vitro* (div) in the presence of complete serum-free expansion medium. After 3 div, cells from protocol 1 (E) and 3 (G) were able to form NS. In contrast, cells from protocol 2 (F) and 4 (H) did not proliferate. Cells from protocol-1 (I) were able to form secondary NS after a 1:1 passage and 7 div, whereas cells from other protocols progressively died (J-L). Scale bar 100μm.

After the cryopreservation process, MGE-derived cells should keep intact their intrinsic properties as progenitors. To verify this, we cultured the cryopreserved cells as NS to assess their proliferation and self-renewal capacity, and whether they remain as undifferentiated progenitors under *in vitro* expansion conditions. Interestingly, we observed marked differences in cell proliferation and ability to form NS depending on the cryopreservation protocol. Thus, progenitors from protocol-1 formed NS after three days in vitro (div) without difficulty (Fig 3A and 3E). This process was repeated in successive culture passages (Fig 3I), confirming their self-renewal capability. In contrast, cells from protocol-2 never formed NS, even after maintaining them in culture during 10 days with periodic changes of the NS culture medium (Fig 3B, 3F and 3J). This result is in concordance with previous literature [38]. On the other hand, cells from protocol-3 generated NS properly. However, comparing with protocol-1 they formed a lower number of cell spheres (Protocol-1 = 18.5 ± 2.53 spheres / plate; protocol-3 = 9.67 ± 2.19 spheres / plate; p = 0.00872, unpaired Student’s t-test) and they needed longer time to be generated (4 days instead of 3) (Fig 3G). Surprisingly, the culture did not form new NS after 1:1 passage (Fig 3K), indicating that stem cell functionality and their ability to self-renew was affected. Finally, cells from protocol-4 were unable to proliferate and they did not form NS (Fig 3D, 3H and 3L), suggesting that freezing the big spherical structure of the NS may seriously affect their cell viability, as previously reported [32,38].

**Fig 4:**
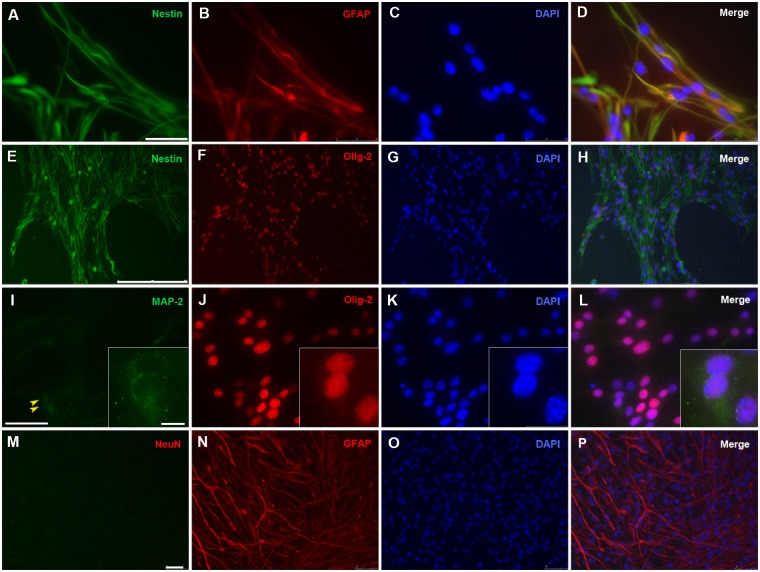
Immunocharacterization of *in toto* cryopreserved MGE-derived tissue in culture. Frozen-thawed *in toto* MGE-derived tissue was grown and allowed to expand on 0.1% gelatin-coated coverslips for 1 month in serum-free expansion complete medium. Cells were fixed and processed for immunocytochemical staining to identify different cellular phenotypes using a combination of markers. (A-D) nestin (green) in combination with glial fibrillary acid protein (GFAP, red) as early markers expressed by immature precursors; (E-H) nestin (green) and the presence of Olig-2 (red), as early specific markers for MGE region precursors. (I-L) MAP-2 (green) for early neuronal marker, the inset shows a higher magnification of the two cells pointed by arrowheads in which a low level accumulation of MAP-2 is detected; (M-P) Neu-N (green) as mature neuronal marker and GFAP (red) as astrocytic marker; 4'-6'-Diamidino-2-phenylindole (DAPI) stain (blue) was used to visualize nuclei. Scale bars in A, I, and M: 50μm; in E: 250μm; in the inset: 10μm.

Given the good results obtained with protocol-1 regarding proliferation and self-renewal parameters, we focus our efforts on this procedure to further characterize their effects on the undifferentiation status of the progenitors and the preservation of their MGE region identity *in vitro*. For this, frozen MGE tissue following protocol-1 was culture on gelatin-pretreated flasks for adhesion, and grew in expansion complete medium in the presence of growth factors (for details see M&M). Immunocytological characterization was performed after 4–6 weeks in culture with antibodies against specific markers for neural progenitors (Nestin + GFAP), astrocytic linage (GFAP), early neuronal lineage (MAP-2), mature neurons (NeuN), oligodendrocytic lineage (NG2), and also for MGE region-specific marker (Olig-2). Co-localization results for nestin and GFAP confirmed that practically the whole culture was undifferentiated and constituted by neural progenitors (Fig 4A–4D). We quantified a 98.7 ± 0.4% of nestin and GFAP double positive cells in culture (progenitors), whereas only a 1.1 ± 0.6% was nestin negative and GFAP positive (mature astrocytes). In concordance, cultures were also negative for NG2 expression (data not shown). In contrast, a small percentage of cells were positive for MAP-2 (5.1 ± 2%, Fig 4I). The labeling was never found in the cellular processes, but concentrated in the soma (arrowheads in Fig 4I and inset). This suggests that few progenitors may be escaping their stemness condition and they are preparing to differentiate into neurons by accumulating MAP-2. No NeuN immunoreactivity was observed in the cultured cells, indicating the absence of mature neurons (Fig 4M). These results suggest that protocol-1 properly maintain the stemness progenitor properties *in vitro*, one of the main requirements for the development of a cell amplification system in culture. Finally, we also found an almost complete colocalization of Olig-2 with nestin in the cultures (Fig 4E–4H). This positive result reflexes a good practice in the MGE tissue dissection and lack of contamination from adjacent areas. It also suggests that cells in culture kept native identity from the region they were isolated.

### *In vivo* characterization of cryopreserved GABAergic precursor after transplant

**Fig 5:**
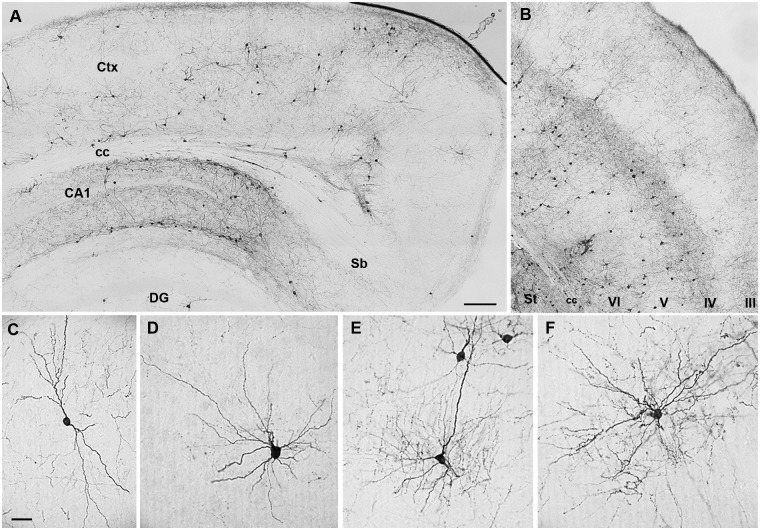
Distribution and differentiation of *in toto* cryopreserved MGE-derived precursors after transplantation. (A) Detection of grafted cells by immunohistochemistry against GFP in the neocortex (Ctx) and hippocampus four weeks after the transplant. (B) Note the wide distribution of cryopreserved grafted cells in multiple cortical layers and the dark background due to dendrite concentration in layer IV. (C-F) MGE-derived cells in the cortex differentiated into neurons presenting typical morphology of interneuron subtypes e.g., bitufted (C), multipolar (D), chandelier cells with synaptic boutons resembling candlesticks (E), or basket cells (F). In hippocampus, grafted cells accumulated in CA1 and dentate gyrus (DG). Corpus callosum (cc); cortical layers (III-VI); striatum (St); subiculum (Sb). Scale bars for A and B: 200μm; in C for D-F: 25μm.

Preservation of *in vivo* migration ability of the MGE-derived precursor and their subsequent differentiation into GABAergic interneurons after transplantation were checked to ensure an appropriate cryopreservation protocol. Thus, in a series of intracerebral transplants in neonate WT mice (n = 6), we verified whether cryopreserved cells from protocol-1 were able to maintain the same properties as when they are isolated from the embryonic brain and immediately transplanted. MGE explants from GFP^+^ embryos were cryopreserved and one month later grafted in parallel to fresh isolated MGE-GFP^+^ cells. Transplanted mice were sacrificed 4 weeks later, time enough for complete maturation and differentiation of grafted cells [12,13]. Survival rate for cryopreserved cells after transplantation was 17.39 ± 1.27%, within the range of previous results from our group and others [13], and not significantly different from their fresh isolated counterparts (18.63 ± 2.08%). Cryopreserved cells from protocol-1 spread throughout the cortex, covering an average maximum distance of 3.85 ± 0.5 mm in both directions of the antero-posterior axe, similar to the migration distances reported for fresh-isolated cells in previous works [9, 15, 16]. They also showed mature interneuron morphology as previously published [9], with long and complex branching typical of interneurons in the adult brain (Fig 5).

To further characterize the acquisition of a fully mature dendritic arborization we performed a Sholl’s analysis [37], a basic method (see M&M section) to study different dendritic properties such as the wealth of branches, the dendritic arborization size, etc. A comparative of the different parameters with the fresh-isolated cells can be found in Table 2. No significant differences were observed, but for RI value. These results strongly suggest that protocol-1 cryopreservation process has minimal effects on the interneuron morphology acquisition by MGE precursors.

**Table 2:** Morphological analysis of MGE-derived cells after transplantation.

*Sholl Analysis*	Fresh-isolated Cells	Cryopreserved Cells
**Nr (μm**	186,50 ± 11,92	185,88 ± 10,33
**Nav (μm)**	10,48 ± 0,99	9,04 ± 0,52
**Area under curve (μm**^**2**^**)**	1917,18 ± 224,51	1711,88 ± 140,60
**Nm (μm)**	18,83 ± 1,64	15,57 ± 1,66
**K**	-5,10 ± 0,11	-5,33 ± 0,12
**RI**	15,84 ± 1,69	7,29 ± 0,97***

Sholl Analysis of cryopreserved (n = 36) and fresh-isolated (n = 34) MGE cells was performed four weeks after their transplant in the neonatal (P3-5) cerebral cortex (n = 8). Data expressed as mean ± SEM. Statistical analysis: unpaired Student's t-test (*** = p<0.001).

**Fig 6:**
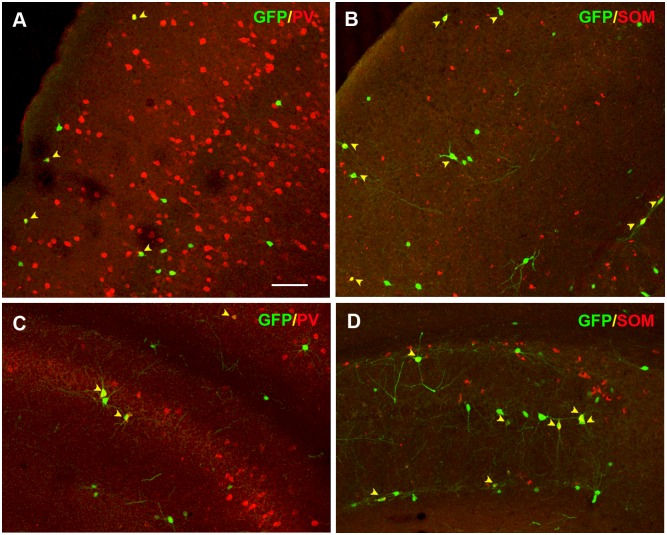
Interneuron sub-type differentiation of MGE-derived *in toto* cryopreserved cells after transplantation. Immunohistochemical colocalization of MGE *in toto* cryopreserved GFP^+^ cells with the interneuron subtype markers PV (A, C) and SOM (B, D) in the cerebral cortex (A-B) and hippocampus (C-D). Arrowheads show double-positive cells. Scale bar 100μm.

Finally, to ensure the maturation at molecular level of the grafted cryopreserved cells we performed an immunohistochemical analysis with different interneuron-subtype specific markers, such as PV, SOM, CR and CB that comprise almost the totality of the cortical and hippocampal GABAergic interneurons (Fig 6). Counts for double positive cells for GFP and each of the interneuron subtype markers are presented in Table 3. Interestingly, the results, compared with previously published quantifications [12,13], showed that cryopreserved precursors differentiated *in vivo* similarly to fresh-isolated MGE cells and kept the same subtype proportions that can be found during normal cortical formation. This suggests that the commitment and capability to mature into different interneuron subtypes are not affected by the *in toto* cryopreservation process.

**Table 3:** Differentiation of MGE-derived *in toto* cryopreserved precursors.

Interneuron Subtypes	Hippocampus	Cortex
**Parvalbumin**	31,08 ± 4,27%	30,9 ± 4,15%
**Somatostatin**	35,93 ± 4,54%	31,38 ± 4,32%
**Neuropeptide Y**	17,15 ± 2,50%	19,95 ± 3,44%
**Calretinin**	10,35 ± 0,95%	1,51 ± 0,3%
**Calbindin**	8,35 ± 0,95%	14,45 ± 1,3%

Thawed cells were immediately grafted into the neonatal brain (P3-5) and quantified 4 weeks after the transplantation (n = 6). Values represent percentages of double positive cells for GFP and each marker in the total population of graft-derived GFP^+^ cells, expressed as mean ± SEM.

On the other hand, in concordance with previous results [12], we also observed GFP^+^ endothelial cells (7.63 ± 2.71%) forming blood vessels just around the injection site, and some oligodendrocytes (2.26 ± 0.86%) close to the injection area. Finally, indicate that, in terms of safety, we never observed any transformation or tumor formation derived from GFP^+^ cryopreserved cells, even a year and a half after transplantation.

## Discussion

Neuronal precursor transplantation is a promising therapeutic strategy for brain repair and treatment of diverse neuropathologies. The accessibility to a permanent source of neuronal progenitors is essential for the clinical application of these cell-based therapies. Biobanks, in where to cryopreserve tissue or neuronal precursors, can meet this need as reliable and constant cell suppliers [39]. However, appropriate cryopreservation protocols are necessary to minimize the effects on cell viability and ensure the correct neuronal progenitor storage. In addition, it may be convenient an amplification of the precursors to increase their availability, keeping always intact their intrinsic properties of migration, proliferation, and differentiation.

The work presented here describes a cryopreservation protocol for MGE-derived GABAergic precursor, an important source of cells with remarkable properties for their application in cell therapy against interneuron-related pathologies such as epilepsy, infantile encephalopathies, schizophrenia, autism or AD [6–9]. We tested four different cryopreservation conditions (see M&M), using 10% Me_2_SO as cryoprotectant in all of them, since in the literature always appears as the most extendedly used and it renders the best results for neuronal cells [23–29]. Our analysis strongly suggests that *in toto* cryopreservation of MGE explants (protocol-1) would be the optimal preservation system to keep intact the interneuron precursor properties for cell transplantation, together with a high cell viability and yield. Comparing with the other three protocols, it offered the highest cell recovery (≈70%) with a cellular viability close to the 80%. These percentages are higher than the usual results found in the literature for individual neurons, where reported values for viability are around the 60–70% [27–29,34].

Differences in cell yield and viability among the tested protocols may be due to the cryoprotectant cytotoxic side-effects [19,21], in our case Me_2_SO. Its cryoprotective mechanism depends on the ability and speed to permeate through the cell membrane during cooling to balance the intra- and extra-cellular osmotic pressure. However, high concentrations of Me_2_SO inside the cell can lead to toxicity [21,23]. The whole MGE explant structure may slow the Me_2_SO penetration into the cells during cryopreservation comparing with dissociated individual cells, what may reduce its concentration in the cell and result in a good balance between permeation and concentration, minimizing the cytotoxic effects and maximizing cryoprotection. In contrast, the mechanical tissue dissociation in other protocols may perturb or damage the progenitor cell membrane and allow a faster entrance of the Me_2_SO, exposing the cell to a higher concentration that may be toxic before freezing takes place [22,40,41]. In this regard, it is interesting to note that post-cryopreservation viabilities of dissociated cells (protocols 2 and 3) are lower than those from whole explants or NS (protocols 1 and 4, respectively). We also observed important differences in the cell recovery yield among protocols. A significant reduction in the number of retrieved cells was detected in those protocols with an *in vitro* amplification step (Fig 2). This suggests that cultured MGE-derived cells are more susceptible to Me_2_SO cytotoxic effects than when they are recently isolated from the embryonic brain. In addition, cells in NS culture are not protected by the extracellular matrix present in the explants or in recently dissociated cells. This absence of a protective cover may facilitate their lysis and also the Me_2_SO permeation during manipulation and freezing.

Protocol-1 was also revealed as the best condition to preserve the capacity of the MGE-precursors to form NS, their *in vitro* long lasting functionality, and self-renewal. *In toto* cryopreserved MGE-explants exhibited a fast *in vitro* growth, suggesting that the transient amplifier cells were intact, allowing a prompt proliferation. Long term stem cells should be also preserved with this protocol, as formation of secondary NS was easily achieved after multiple passages along the time. This may be explained by the maintenance of paracrine signals and cell-cell contacts in the cryopreserved explants. It has been shown that a close cell-cell interaction and the notch signaling are necessary to keep the progenitor activity and stemness in NS cultures [42,43]. The *in toto* structure may be facilitating the cellular interactions and preserving the formation of a notch microenvironment that promote the posterior stem cell proliferation and the preservation of the long-term stem cells. In contrast, when the cellular association is completely broken down, as in the single-cell suspension protocols that we tested, the cell viability after cryopreservation is affected. Interestingly, cellular interaction and sphere diameter have also shown to be relevant parameters in the survival and viability of neural progenitors cultured as NS [32,38,44]. This may explain why in protocols 3 and 4 took longer to form NS or their self-renewal activity was impaired.

The *in vivo* results after transplantation confirmed that *in toto* cryopreservation of MGE-derived precursors keeps intact their potency to differentiate into several interneuron subtypes. This is especially relevant for their application in cell-based therapy. Migration and differentiation properties were similar to precursors recently isolated from the MGE. The cryopreserved transplanted cells showed mature morphology, with long and complex ramifications characteristics of interneurons, and when compared to fresh-isolated grafted cells they showed a similar dendritic arborization, confirmed by Scholl analysis. No differences in the main measured parameters for dendrite extension, cell size or form were observed. Finally, we found that cryopreserved cells were able to survive in the adult brain for over a year without signs of tumor formation (data not shown), demonstrating that even with a freezing process prior to transplantation they remain highly stable and safe.

The cryopreservation procedure we have stablished here for murine GABAergic neuronal precursors may represent a starting point for the development of new protocols to cryopreserve human neural stem cells and human GABAergic progenitor cells for regenerative medicine. Adaptation to human samples will be easy since no products of animal origin, such as serum, were included in the protocol to avoid the risk of zoonosis in a future. Nonetheless, further research should be performed to confirm this protocol can be applied to human samples. Taken together, the data suggest that MGE cryopreserved *in toto* is an excellent system for proper cryopreservation of murine MGE-derived GABAergic precursors. We hope these studies will help to ease the clinical implementation of cell-based therapies against an important group of interneuron-related disorders.
